# Prognostic Factors for Mortality in ICU Patients With Healthcare-Associated Infections: A Five-Year Institutional Analysis

**DOI:** 10.7759/cureus.105865

**Published:** 2026-03-25

**Authors:** Arif Timuroglu, Burcu Caliskan Demirkiran

**Affiliations:** 1 Anesthesiology and Reanimation, Dr. Abdurrahman Yurtaslan Ankara Oncology Hospital, Ankara, TUR; 2 Infectious Diseases, Dr. Abdurrahman Yurtaslan Ankara Oncology Hospital, Ankara, TUR

**Keywords:** central line-associated bloodstream infection, critical care, healthcare-associated infections, oncology intensive care, urinary tract infections, ventilator-associated pneumonia

## Abstract

Background

Healthcare-associated infections (HAIs) represent a major challenge in intensive care units (ICUs), leading to increased morbidity, prolonged hospitalization, and high mortality. Critically ill oncology patients are particularly vulnerable because of immunosuppression and frequent exposure to invasive devices.

Objective

This study aims to describe the epidemiology and microbiological profile of HAIs among oncology ICU patients over a five-year period and to identify independent risk factors for ICU mortality using multivariable analysis.

Methods

We conducted a single-center retrospective cohort study in the anesthesiology ICU of a tertiary oncology hospital between January 1, 2020, and December 31, 2024. Among 1,766 ICU admissions, 259 adult patients who developed at least one HAI (343 episodes in total) and met the inclusion criteria were analyzed. Data on demographics, comorbidities, infection characteristics, microbiology, and outcomes were retrieved from the hospital infection control surveillance system and medical records. Comparative analyses between survivors and non‑survivors were performed, and a parsimonious multivariable logistic regression model was constructed to identify independent predictors of ICU mortality. Model performance was assessed by the area under the receiver operating characteristic (ROC) curve (AUC) and the Hosmer-Lemeshow test.

Results

The median ICU length of stay (LOS) was 29 days (interquartile range (IQR): 17-49), and ICU mortality was 81.1% (n = 210). Pneumonia (including ventilator‑associated and hospital‑acquired pneumonia) was the most common HAI (45.1%, n = 117), followed by central line‑associated bloodstream infection (CLABSI) (35.1%, n = 91) and catheter‑associated urinary tract infection (CAUTI) (11.6%, n = 30). *Klebsiella* spp. (37.6%, n = 129) and *Acinetobacter baumannii* (23.9%, n = 82) were the predominant pathogens. In the final multivariable model, higher age (adjusted odds ratio (aOR): 1.03 per year, 95% confidence interval (CI): 1.01-1.05, p = 0.014), presence of malignancy (aOR: 3.01, 95% CI: 1.49-6.10, p = 0.002), and pneumonia compared with other infection sites (aOR: 2.89, 95% CI: 1.41-5.94, p = 0.004) were independently associated with increased ICU mortality. The model showed acceptable discrimination (AUC: 0.741) and good calibration (Hosmer-Lemeshow, p = 0.363). In a sensitivity analysis restricted to ventilator‑associated pneumonia cases only, pneumonia remained independently associated with ICU mortality (aOR: 2.71, p = 0.007).

Conclusion

Among ICU patients with HAIs, mortality was very high. Older age, underlying malignancy, and pneumonia were the main independent prognostic factors associated with ICU mortality, particularly in the context of prolonged device exposure and highly virulent Gram‑negative pathogens. These findings highlight the need for intensified, site‑specific infection prevention strategies and strengthened antimicrobial stewardship in oncology ICUs, while recognizing that residual confounding related to illness severity and antimicrobial resistance patterns cannot be fully excluded.

## Introduction

Healthcare-associated infections (HAIs) represent a critical global challenge in intensive care units (ICUs), substantially increasing morbidity, mortality, and healthcare costs [1]. Critically ill patients are particularly vulnerable due to hemodynamic instability, impaired immune responses, and the frequent use of invasive procedures such as mechanical ventilation and central venous catheterization [2]. While global reports indicate that 7%-15% of hospitalized patients develop at least one HAI, this prevalence is significantly higher in the ICU setting [3].

The dynamic epidemiology of HAIs is driven by evolving patient demographics, resistance patterns, and regional infection control practices [4]. Although international studies such as EPIC II and EUROBACT provide a global perspective, local data remain essential for tailoring empirical antibiotic therapies and improving clinical outcomes [1,4,5]. This is especially critical in specialized centers, such as oncology-predominant hospitals, where patients present with unique risk factors including immunosuppression and complex surgical histories.

In Türkiye, there is a paucity of contemporary data specifically addressing prognostic factors for mortality among ICU patients with healthcare-associated infections in oncology-predominant intensive care units.

This study aims to analyze the healthcare-associated infections and mortality rates in an anesthesia ICU of a major oncology hospital over a five-year period. By identifying the prognostic factors associated with mortality, we seek to contribute to the optimization of infection management and patient survival in similar clinical environments. We hypothesized that specific clinical factors, including advanced age, underlying malignancy, and the site of infection, would serve as independent predictors of mortality among patients who develop HAIs in an oncology-focused intensive care setting.

## Materials and methods

Study design and setting

This study was designed as a single-center, retrospective, observational cohort study conducted at the Anesthesiology Intensive Care Unit (ICU) of Dr. Abdurrahman Yurtaslan Ankara Oncology Training and Research Hospital. The study period spanned five years, from January 1, 2020, to December 31, 2024. The study protocol was initially reviewed and approved by the Institutional Education and Research Committee on February 10, 2026. Final formal approval was granted by the Non-interventional Clinical Research Ethics Committee (approval date: March 5, 2026; decision number: 2026-03/41). Data extraction from the existing electronic records of the Hospital Infection Control Committee and subsequent statistical analysis were initiated only after all institutional and ethical approvals were secured. Due to the retrospective nature of the data collection and the use of de-identified surveillance records, the requirement for informed consent was waived by the ethics committee. All procedures were carried out in compliance with the ethical standards set forth in the Declaration of Helsinki.

Inclusion and exclusion criteria

The study population was derived from a total of 1,766 admissions recorded in the ICU during the specified five-year period. To be eligible for inclusion, patients had to be aged 18 years or older and must have developed at least one HAI during their ICU stay. In accordance with the Centers for Disease Control and Prevention (CDC) and National Healthcare Safety Network (NHSN) criteria, only infections occurring 48 hours or more after ICU admission were considered [6].

Conversely, several exclusion criteria were applied to ensure the integrity of the prognostic analysis. Patients with an ICU stay of less than 48 hours were excluded from the study, as were those who presented with community-acquired infections without developing a subsequent HAI. Furthermore, patients with incomplete or insufficient medical records regarding their infection parameters or clinical outcomes were also excluded. Following the application of these criteria, a final cohort of 259 adult patients was enrolled in the analysis.

Definition of healthcare-associated infection

Healthcare-associated infection was defined as an infection that was neither present nor in the incubation phase at the time of ICU admission, occurring 48 hours or more after the patient’s entry into the intensive care unit. The diagnosis of HAI was established based on clinical, laboratory, and microbiological evidence in accordance with internationally recognized surveillance criteria [6,7]. Infection types were classified into several categories, including ventilator-associated pneumonia (VAP), central line-associated bloodstream infection (CLABSI), catheter-associated urinary tract infection (CAUTI), surgical site infection, and other infections acquired within the ICU.

Definition of hospital-acquired pneumonia (HAP)

Hospital-acquired pneumonia (HAP) was defined as a pulmonary infection developing 48 hours or more after hospital admission in patients not undergoing invasive mechanical ventilation at the time of diagnosis. Diagnosis was established when new or worsening infiltrates were identified on chest imaging, accompanied by at least one systemic inflammatory sign, including fever exceeding 38°C, hypothermia below 36°C, leukocytosis of 12,000/mm³ or greater, or leukopenia of 4,000/mm³ or less, and at least one respiratory manifestation, such as purulent or increased respiratory secretions or deterioration in oxygenation [7].

Definition of ventilator-associated pneumonia (VAP)

In the present study, VAP was defined as pneumonia developing in patients who had been receiving invasive mechanical ventilation through an endotracheal or tracheostomy tube for a minimum of 48 hours. Confirmation of the diagnosis required the identification of new or worsening pulmonary infiltrates on chest imaging, along with at least one systemic indicator of infection, namely, fever above 38°C, hypothermia below 36°C, leukocytosis of 12,000/mm³ or above, or leukopenia of 4,000/mm³ or below, and at least one respiratory sign, such as purulent tracheal secretions, an increase in respiratory secretions, or a decline in oxygenation status. Microbiological evaluation of lower respiratory tract samples, including endotracheal aspirates or bronchoalveolar lavage, was performed when available and was considered supportive but not mandatory for diagnosis. These criteria are consistent with established international definitions of ventilator-associated pneumonia [6,7].

Data collection

Patient data were retrospectively obtained from the hospital information management system, infection control committee records, microbiology laboratory reports, and medical charts. All data were recorded by the investigators using a standardized data collection form prepared prior to the study.

Comorbid conditions and relevant clinical risk factors were documented, including diabetes mellitus (DM), hypertension (HT), chronic obstructive pulmonary disease (COPD), chronic kidney disease, coronary artery disease, heart failure, neurological disorders, and malignancy. In addition, exposure to invasive interventions, such as mechanical ventilation, central venous catheter insertion, total parenteral nutrition, the presence of surgical drains, and receipt of blood transfusions, was also recorded.

Infection‑related data included the date on which the infection was diagnosed, the anatomical site or type of infection, such as pneumonia, bloodstream infection (BSI), urinary tract infection, surgical site infection, or catheter‑associated infection, and the number of days between ICU admission and the onset of infection. Microbiological isolates were also recorded, including *Acinetobacter baumannii*,* Klebsiella* sp.,* Pseudomonas aeruginosa*,* Escherichia coli*,* Enterococcus *sp.,* Candida *sp., coagulase‑negative staphylococci,* Stenotrophomonas maltophilia*,* Serratia *sp.,* Proteus* sp., *Enterobacter* sp., *Burkholderia* sp., and other identified pathogens.

For patients with more than one HAI episode, only the first documented HAI was used for all comparative and regression analyses to maintain patient-level independence and avoid survivor bias. Subsequent episodes were included only for descriptive microbiological statistics. Using the first HAI minimizes the risk that late-onset or terminal infections (which are more common in non-survivors) artificially inflate the association between infection type and mortality.

Standardized illness severity scores such as the Acute Physiology and Chronic Health Evaluation II (APACHE II) and Sepsis-related Organ Failure Assessment (SOFA) could not be reliably calculated for the cohort due to incomplete or inconsistently timed recording of several required physiological and laboratory parameters in this retrospective dataset; therefore, these scores were not included in the multivariable model.

Notably, as a specialized tertiary oncology center, our institution was designated as a non-COVID facility during the pandemic to protect the immunocompromised patient population. Patients with a diagnosis of COVID-19 were not admitted to the anesthesia ICU, and any patient testing positive for SARS-CoV-2 was transferred to a pandemic-specific hospital. Thus, the study cohort represents a population unaffected by COVID-19-related clinical complications.

Missing data were handled using a complete case analysis approach. A small proportion of patients (approximately 2.5% of the initially eligible ICU admissions) had missing critical variables (e.g., infection site or outcome) and were therefore excluded from the final multivariable model. Given this low level of missingness and the fact that missingness was primarily due to incomplete documentation rather than a specific clinical pattern, we considered that formal imputation was not necessary and unlikely to change the main findings.

The primary outcome of this study was all-cause ICU mortality. Secondary outcomes included the distribution of HAI types (pneumonia, CLABSI, CAUTI, and BSI), the microbiological profile of causative pathogens, and the ICU length of stay (LOS). These secondary outcomes were analyzed descriptively to characterize the epidemiological and clinical burden of HAIs in the study population.

Statistical analysis

All statistical procedures were carried out using the Statistical Package for the Social Sciences (SPSS) software package version 24.0 (IBM Corp., Armonk, NY). Continuous data were reported as mean ± standard deviation or as median with interquartile range (IQR) or minimum-maximum values, depending on the distribution. Categorical variables were summarized as frequencies and percentages.

For descriptive analyses, frequencies, percentages, means, medians, and measures of dispersion were calculated for all patient- and infection-related characteristics.

To identify factors associated with ICU mortality, patients were stratified into survivor and non-survivor groups for bivariate comparisons. For continuous variables, between-group differences were evaluated using the Student's t-test when data followed a normal distribution, or the Mann-Whitney U test when normality could not be assumed. Comparisons of categorical variables were conducted using the chi-square test or Fisher's exact test, depending on the expected cell frequencies.

Variables demonstrating a statistically significant association with mortality at p < 0.10 in the bivariate analyses were subsequently subjected to univariable logistic regression analysis, with adjusted odds ratios (aORs) and corresponding 95% confidence intervals (CIs) calculated for each variable.

To identify the independent predictors of ICU mortality, a multivariable logistic regression model was constructed using the "enter" method. To ensure a robust model and maintain an adequate events-per-variable (EPV) ratio (approaching the recommended threshold of 10), a parsimonious model was developed by including the most clinically and statistically significant variables (p < 0.10 in univariable analysis), including age, malignancy status, invasive mechanical ventilation, and a binary infection site variable (pneumonia versus non-pneumonia).

Because the number of HAP cases was very small (n = 4), VAP and HAP were combined into a single pneumonia category for the primary multivariable analysis to avoid sparse data problems. In an additional sensitivity analysis restricted to VAP cases only, pneumonia remained independently associated with ICU mortality, supporting the robustness of our findings.

To ensure model stability and avoid overfitting, a parsimonious approach was adopted, and multicollinearity among predictors was assessed using variance inflation factors (VIF). All VIF values were <2.0, indicating no significant multicollinearity.

Variables with a p-value < 0.10 in the univariable analysis were subsequently entered into a multivariable logistic regression model. Independent prognostic factors were identified using the enter method. The discriminative ability of the final multivariable model was evaluated by calculating the area under the receiver operating characteristic (ROC) curve (AUC). Model fit was assessed using the Hosmer-Lemeshow goodness-of-fit test.

Additionally, a Cox proportional hazards model was constructed as a time-to-event analysis to evaluate the association between clinical variables and the hazard of ICU mortality, with the time origin defined as the date of ICU admission.

For all statistical tests, a two-tailed p-value of less than 0.05 was considered to indicate statistical significance.

## Results

Baseline characteristics of the study population

**Figure 1:**
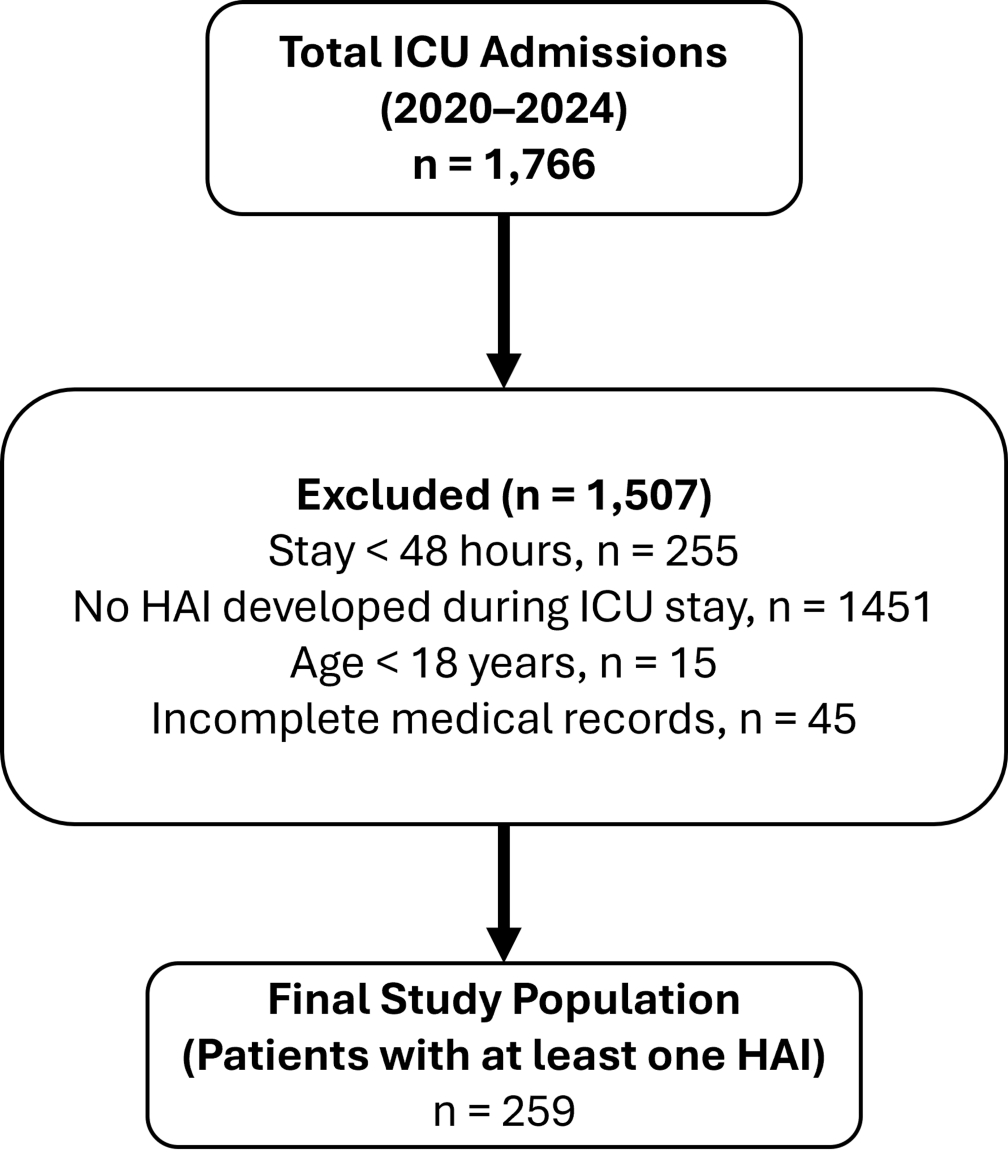
Flow diagram of patient selection from all ICU admissions to the final cohort of 259 patients with healthcare-associated infections Of 1,766 total ICU admissions recorded between 2020 and 2024, 1,507 patients were excluded (255 with ICU stay < 48 hours, 1,451 with no HAI during ICU stay, 15 aged <18 years, and 45 with incomplete medical records). The final study population comprised 259 adult patients who developed at least one HAI during their ICU stay. ICU: intensive care unit, HAI: healthcare-associated infection

A total of 1,766 admissions were recorded in the anesthesiology ICU during the five-year study period. From this population, 259 adult patients who met the inclusion criteria were enrolled in the final analysis. Because some patients experienced more than one healthcare-associated infection during their ICU stay, these 259 patients accounted for a total of 343 distinct HAI episodes. The study population had a median age of 69 years, with an IQR spanning from 58 to 78 years. The median hospital length of stay was 38 days (IQR: 24-61), and the median ICU length of stay was 29 days (IQR: 17-49). The median time from ICU admission to infection diagnosis was 24 days (IQR: 15-35) (Table 1). The selection process from the initial ICU admissions to the final analytic cohort of 259 patients with HAIs is summarized in a flow diagram (Figure 1).

**Table 1:** Demographic and clinical characteristics of the patients (N = 259) Data are presented as median (interquartile range) for continuous variables and number (%) for categorical variables. IQR: interquartile range, ICU: intensive care unit, GI: gastrointestinal, CPR: cardiopulmonary resuscitation, COPD: chronic obstructive pulmonary disease Note: Patients could have more than one comorbidity or intervention; therefore, the sum of these categories exceeds the total number of patients (N = 259).

Characteristics	Values
Age and length of stay	
Age, years, median (IQR)	69 (58-78)
Hospital length of stay, days, median (IQR)	38 (24-61)
ICU length of stay, days, median (IQR)	29 (17-49)
Day of infection diagnosis, days from ICU admission, median (IQR)	24 (15-35)
Gender, number (%)	
Female	131 (50.6)
Male	128 (49.4)
ICU outcome, number (%)	
Non-survivor (mortality)	210 (81.1)
Survivor	49 (18.9)
Admission diagnosis, number (%)	
Respiratory failure	194 (74.9)
Sepsis	27 (10.4)
Acute renal failure	11 (4.2)
Trauma	9 (3.5)
Others (post-CPR, GI bleeding, chronic liver disease, etc.)	18 (7.0)
Comorbidities, number (%)	
Malignancy (total)	126 (48.7)
Solid tumor	95 (36.7)
Hematologic malignancy	31 (12.0)
Hypertension	57 (22.0)
Diabetes mellitus	40 (15.4)
COPD	23 (8.9)
Neurological disease	22 (8.5)
Coronary artery disease	18 (6.9)
Chronic renal failure	14 (5.4)
Heart failure	10 (3.9)
Type of healthcare-associated infection, number (%)	
Ventilator-associated pneumonia	113 (43.6)
Central line-associated bloodstream infection	91 (35.1)
Catheter-associated urinary tract infection	30 (11.6)
Bloodstream infection	21 (8.1)
Hospital-acquired pneumonia	4 (1.5)
Risk factors and interventions, number (%)	
Invasive mechanical ventilation	218 (84.2)
Central venous catheterization	218 (84.2)
Total parenteral nutrition	60 (23.2)
Blood transfusion	54 (20.8)
Presence of surgical drain	27 (10.4)

The cohort was evenly distributed by gender (female: 50.6%, n = 131). ICU mortality was high, with 81.1% (n = 210) of patients classified as non-survivors. The most common admission diagnosis was respiratory failure (74.9%, n = 194), followed by sepsis (10.4%, n = 27). Nearly half of the patients had an underlying malignancy (48.7%, n = 126), including solid tumors (36.7%, n = 95) and hematologic malignancies (12.0%, n = 31).

VAP was the most frequent HAI (43.6%, n = 113), followed by CLABSI (35.1%, n = 91) and CAUTI (11.6%, n = 30). The majority of patients required invasive mechanical ventilation (84.2%, n = 218) and central venous catheterization (84.2%, n = 218) (Table 1).

Since individual patients could present with multiple comorbidities and undergo various clinical interventions simultaneously, the total counts within the "Comorbidities" and "Risk factors and interventions" categories in Table 1 exceed the overall study population of 259.

Comparison between survivors and non-survivors

The comparative analysis of clinical and demographic characteristics between survivors (19%, n = 49) and non-survivors (81%, n = 210) revealed several key differences (Table 2). While the median age was slightly higher in the non-survivor group (69 years versus 67 years), this difference did not reach statistical significance (p = 0.216). Notably, survivors had significantly longer clinical courses, with a median hospital stay of 52 days compared to 36 days in non-survivors (p < 0.001), and a median ICU stay of 47 days versus 27 days (p < 0.001). The timing of infection diagnosis from ICU admission was nearly identical in both groups, occurring at a median of approximately 24-25 days (p = 0.849). The significantly longer hospital and ICU stay observed among survivors (p < 0.001) likely reflect survivorship bias, as non-survivors had a shorter "time at risk" to accumulate length of stay, rather than indicating a protective clinical effect of prolonged hospitalization.

**Table 2:** Comparison of clinical characteristics between survivors and non-survivors (N = 259) Data are presented as median (IQR) for continuous variables and number (%) for categorical variables. P-values are derived from the Mann-Whitney U test for continuous variables and the chi-square test (or Fisher's exact test when appropriate) for categorical variables. IQR: interquartile range, ICU: intensive care unit, BSI: bloodstream infection, HAP: hospital-acquired pneumonia, COPD: chronic obstructive pulmonary disease

Characteristics (age and length of stay)	Survivors (n = 49)	Non-survivors (n = 210)	p-value
Age, years, median (IQR)	67 (51-78)	69 (59-79)	0.216
Hospital length of stay, days, median (IQR)	52 (36-86)	36 (22-56)	<0.001
ICU length of stay, days, median (IQR)	47 (22-72)	27 (16-42)	<0.001
Day of infection diagnosis, days from ICU admission, median (IQR)	25 (13-37)	24 (15-35)	0.849
Gender, number (%)			0.098
Female	30 (61.2)	101 (48.1)	
Male	19 (38.8)	109 (51.9)	
Admission diagnosis, number (%)			0.042
Respiratory failure	32 (65.3)	162 (77.1)	
Sepsis	5 (10.2)	22 (10.5)	
Others	12 (24.5)	26 (12.4)	
Type of healthcare-associated infection, number (%)			<0.001
Ventilator-associated pneumonia	13 (26.5)	100 (47.6)	
Central line-associated bloodstream infection	16 (32.7)	75 (35.7)	
Catheter-associated urinary tract infection	14 (28.6)	16 (7.6)	
Others (BSI, HAP)	6 (12.2)	19 (9.1)	
Comorbidities, number (%)			
Malignancy (total)	17 (34.7)	109 (51.9)	0.094
Diabetes mellitus	6 (12.2)	34 (16.2)	0.491
Hypertension	10 (20.4)	47 (22.4)	0.764
COPD	2 (4.1)	21 (10.0)	0.190
Chronic renal failure	2 (4.1)	12 (5.7)	0.649
Coronary artery disease	2 (4.1)	16 (7.6)	0.381
Heart failure	2 (4.1)	8 (3.8)	0.929
Neurological disease	3 (6.1)	19 (9.0)	0.508
Interventions, number (%)			
Invasive mechanical ventilation	38 (77.6)	180 (85.7)	0.159
Central venous catheterization	39 (79.6)	179 (85.2)	0.330
Total parenteral nutrition	14 (28.6)	46 (21.9)	0.319
Blood transfusion	12 (24.5)	42 (20.0)	0.486
Presence of surgical drain	8 (16.3)	19 (9.0)	0.133

Regarding admission diagnoses, respiratory failure was the predominant cause in both groups but was more frequently observed among non-survivors (77.1%, n = 162 versus 65.3%, n = 32; p = 0.042). The distribution of HAI types also showed a marked disparity (p < 0.001). VAP was substantially more prevalent among those who died (47.6%, n = 100) than those who survived (26.5%, n = 13). Conversely, CAUTI were significantly more common in the survivor group (28.6%, n = 14 versus 7.6%, n = 16).

In terms of underlying conditions, malignancy was more frequent in the non-survivor group (51.9%, n = 109 versus 34.7%, n = 17), showing a trend toward significance (p = 0.094). Other comorbidities, such as diabetes mellitus and hypertension, were distributed similarly between the two groups. Furthermore, while the requirement for invasive mechanical ventilation and central venous catheterization was high across the entire cohort, no statistically significant differences were found between survivors and non-survivors regarding these interventions or other risk factors such as blood transfusion and total parenteral nutrition.

Multivariable analysis of mortality risk factors

In the final multivariable logistic regression model (Table 3), age, malignancy status, and pneumonia were independently associated with ICU mortality. For every one-year increment in age, the likelihood of ICU mortality increased by 2.5% (aOR = 1.025, 95% CI: 1.005-1.045, p = 0.014). The presence of malignancy remained a strong independent predictor; patients with an underlying malignancy had a threefold higher risk of death compared to those without malignancy (aOR: 3.01, 95% CI: 1.49-6.10, p = 0.002). Furthermore, the site of infection was a critical determinant of survival; patients with pneumonia (including VAP and HAP) had nearly a threefold higher mortality risk compared to those with other types of healthcare-associated infections (aOR: 2.89, 95% CI: 1.41-5.94, p = 0.004). Invasive mechanical ventilation was not independently associated with mortality in the adjusted model (p = 0.132).

**Table 3:** Multivariable logistic regression analysis of factors associated with ICU mortality Data are presented as B, SE, Wald statistics, p-values, and aOR with 95% CI. ICU mortality was the dependent variable (1 = non-survivor, 0 = survivor). For malignancy and pneumonia, "absent" and "no" were used as the reference categories, respectively. The pneumonia category includes both VAP and HAP. Model fit statistics: Omnibus test, p < 0.001; Hosmer-Lemeshow, p = 0.363; Nagelkerke, R² = 0.138; AUC = 0.741 aOR: adjusted odds ratios, CI: confidence intervals, VAP: ventilator-associated pneumonia, HAP: hospital-acquired pneumonia, ICU: intensive care unit, AUC: area under the curve, MV: mechanical ventilation, B: regression coefficients, SE: standard errors

Variable	B	SE	Wald	df	p-value	aOR	95% CI
Age (per year)	0.024	0.010	6.072	1	0.014	1.025	1.005-1.045
Invasive MV (yes versus no)	-0.629	0.417	2.271	1	0.132	0.533	0.235-1.208
Pneumonia (yes versus no)	1.061	0.368	8.326	1	0.004	2.888	1.405-5.937
Malignancy (present versus absent)	1.102	0.359	9.394	1	0.002	3.012	1.488-6.098
Constant	0.209	0.651	0.103	1	0.749	1.232	-

**Figure 2:**
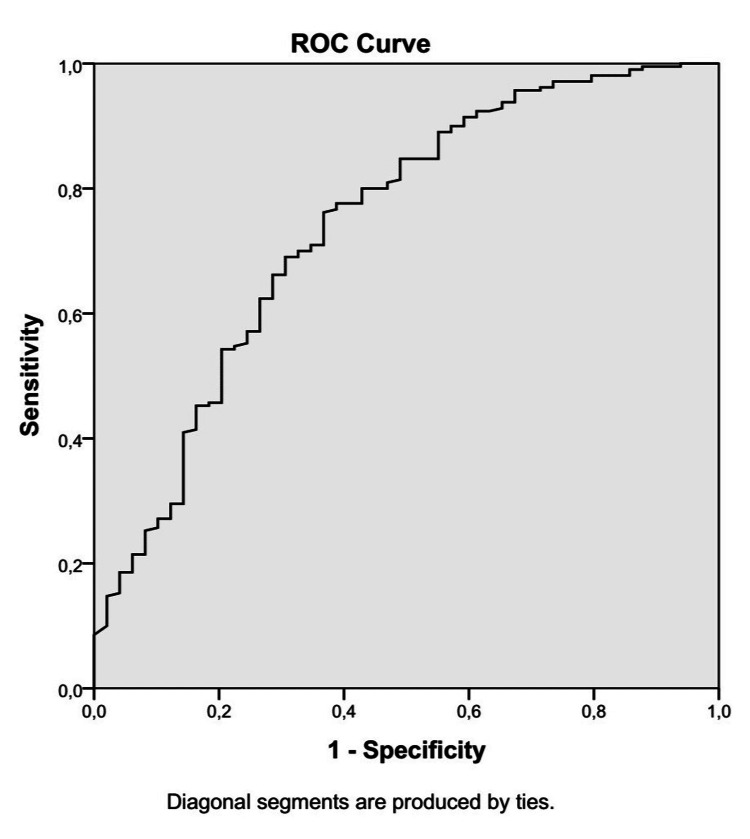
ROC curve for the multivariable logistic regression model predicting ICU mortality The model demonstrated acceptable discriminative performance with an AUC of 0.741. AUC: area under the curve, ROC: receiver operating characteristic, ICU: intensive care unit

The final model demonstrated acceptable discriminative ability, with an area under the receiver operating characteristic (ROC) curve (AUC) of 0.741 and good calibration according to the Hosmer-Lemeshow test (p = 0.363), indicating that the model's predictions were well aligned with the observed outcomes (Figure 2).

A complementary Cox proportional hazards analysis was performed to account for the time-to-event nature of ICU mortality. However, the overall Cox model did not reach statistical significance (χ^2^ = 7.565, df = 4, p = 0.109), and individual covariates, including age, pneumonia, and malignancy, were not independently associated with the hazard of death in this time-dependent analysis. This lack of significance in the Cox model likely reflects the high and relatively uniform mortality rate (81.1%) and the similar timing of events across the cohort, suggesting that the binary outcome (death versus survival) is a more discriminative endpoint in this specific population than the timing of death.

Sensitivity analysis

To assess the robustness of the primary prognostic model, a sensitivity analysis was performed by stratifying the cohort according to malignancy status. In patients without malignancy (n = 133), the multivariable model was statistically significant (χ² = 23.5, p = 0.001; Hosmer-Lemeshow, p = 0.505), with age (aOR: 1.044, 95% CI: 1.017-1.071, p = 0.001) and infection site (p = 0.012) as independent predictors of mortality. Pneumonia was associated with the highest mortality risk in this subgroup, while CAUTI was associated with significantly lower odds of death compared to pneumonia (aOR: 0.119, 95% CI: 0.034-0.424, p = 0.001). In patients with malignancy (n = 126), the model remained significant (χ² = 15.6, p = 0.016; Hosmer-Lemeshow, p = 0.823), with infection site (p = 0.027) and female gender (aOR: 0.274, 95% CI: 0.076-0.995, p = 0.049) as significant predictors. Notably, the protective association of CAUTI relative to pneumonia was consistent across both subgroups (aOR: 0.120, 95% CI: 0.022-0.643, p = 0.013), confirming the robustness of the primary model's infection site findings.

Microbiological profile of healthcare-associated infections

During the study period, 343 HAI episodes were documented among the 259 patients, indicating that 84 additional infection episodes occurred as secondary or recurrent events in the same patient population. The most frequently isolated microorganism was *Klebsiella* spp. (37.6%, n = 129), followed by *Acinetobacter baumannii* (23.9%, n = 82) and *Candida* spp. (14.3%, n = 49) (Table 4). Overall, Gram-negative bacteria predominated.

**Table 4:** Distribution of causative microorganisms in healthcare-associated infections (N = 343 episodes) Data are presented as number (%). Percentages for infection site-specific columns are calculated within each infection type. Some episodes involved polymicrobial infections; therefore, column totals may exceed 100%. VAP: ventilator-associated pneumonia, CLABSI: central line-associated bloodstream infection, CAUTI: catheter-associated urinary tract infection, BSI: bloodstream infection, HAP: hospital-acquired pneumonia Note: The "Total" column includes all identified microorganisms across all 343 healthcare-associated infection episodes. The sum of site-specific counts (VAP, CLABSI, CAUTI, and BSI) may be less than the total count for certain microorganisms (e.g., *Klebsiella* spp. and *Acinetobacter baumannii*) because some isolates were identified in other infection types, such as HAP, which are not displayed as individual columns due to their low frequency.

Microorganism	Total, number (%)	VAP, number (%)	CLABSI, number (%)	CAUTI, number (%)	BSI, number (%)
*Klebsiella* spp.	129 (37.6)	61 (44.5)	38 (29.2)	17 (42.5)	11 (35.5)
Acinetobacter baumannii	82 (23.9)	46 (33.6)	19 (14.6)	4 (10.0)	9 (29.0)
*Candida* spp.	49 (14.3)	1 (0.7)	42 (32.3)	0 (0.0)	6 (19.4)
Pseudomonas aeruginosa	28 (8.2)	21 (15.3)	1 (0.8)	5 (12.5)	1 (3.2)
*Enterococcus* spp.	15 (4.4)	0 (0.0)	11 (8.5)	3 (7.5)	1 (3.2)
Stenotrophomonas maltophilia	14 (4.1)	10 (7.3)	3 (2.3)	1 (2.5)	0 (0.0)
Coagulase-negative staphylococci	12 (3.5)	0 (0.0)	11 (8.5)	0 (0.0)	1 (3.2)
Escherichia coli	11 (3.2)	2 (1.5)	4 (3.1)	4 (10.0)	1 (3.2)
*Enterobacter* spp.	9 (2.6)	4 (2.9)	1 (0.8)	3 (7.5)	0 (0.0)
*Proteus* spp.	8 (2.3)	4 (2.9)	1 (0.8)	3 (7.5)	0 (0.0)
*Serratia* spp.	7 (2.0)	3 (2.2)	3 (2.3)	0 (0.0)	1 (3.2)
Other microorganisms	7 (2.0)	2 (1.5)	5 (3.8)	0 (0.0)	0 (0.0)
Burkholderia cepacia	4 (1.2)	4 (2.9)	0 (0.0)	0 (0.0)	0 (0.0)

The distribution of microorganisms varied by infection type. In VAP, *Klebsiella* spp. (44.5%, n = 61) and *Acinetobacter baumannii* (33.6%, n = 46) were the leading pathogens. In CLABSI, *Candida* spp. (32.3%, n = 42) and *Klebsiella* spp. (29.2%, n = 38) were most frequently identified. CAUTI was predominantly caused by *Klebsiella* spp. (42.5%, n = 17), followed by *Pseudomonas aeruginosa* (12.5%, n = 5). *Enterococcus* spp. and coagulase-negative staphylococci were primarily isolated in bloodstream infections, consistent with their known association with catheter-related infections.

## Discussion

Our study highlights that in an ICU setting with a high prevalence of underlying malignancy, the mortality of patients with HAIs is primarily driven by the type of infection and the patient's oncologic status. The most striking finding was the impact of pneumonia (including both VAP and HAP), which increased the risk of mortality sevenfold compared to patients with CAUTI. This finding aligns with the high-acuity nature of our cohort and suggests that respiratory infections represent the most lethal complication for critically ill patients in this setting. While modern bundle therapies have improved VAP outcomes in general ICUs, our data indicate that pneumonia remains a devastating event, likely due to compromised pulmonary reserves and the high virulence of pathogens encountered in our unit.

Another key finding of our study was that the presence of malignancy independently tripled the risk of ICU mortality (aOR: 2.99). In our cohort, nearly half of the patients (48.7%, n = 126) had an underlying malignancy. While some studies in general ICUs suggest that acute physiological scores are better predictors of outcome than the underlying disease, our results underscore that malignancy itself remains a critical determinant of survival when an infection occurs [8]. This increased risk may be attributed to the baseline immunosuppression associated with cancer, the cumulative effects of prior chemotherapy or radiotherapy, and a potentially diminished physiological reserve to withstand the systemic inflammatory response triggered by a healthcare-associated infection.

Advancing age also emerged as a significant independent predictor of ICU mortality in our multivariable model (aOR: 1.03 per year, p = 0.004). This finding is consistent with the broader literature indicating that elderly patients have diminished physiological reserves and are more vulnerable to severe infections. Our results are further supported by a recent observational study from our institution by Şahin et al., which utilized the Assess Respiratory Risk in Surgical Patients in Catalonia (ARISCAT) risk index in major abdominal cancer surgery [9]. They identified age as the strongest independent predictor of complications, with patients over 80 years old facing a nearly 14-fold increased risk [9]. In our high-acuity oncology ICU, the impact of age likely synergizes with the immunosuppressive effects of malignancy and the virulence of healthcare-associated pathogens.

The overall ICU mortality in our cohort (81.1%, n = 210) is high, reflecting the specific nature of our study population, which consisted exclusively of critically ill oncology patients who developed at least one healthcare-associated infection. Direct comparison with general ICU mortality rates may be misleading, as those cohorts typically include a broader range of admission severities and patients without infections. Mortality in ICU patients with HAIs is known to be substantially higher than in those without such complications [10]. In oncology-focused settings, the combination of baseline immunosuppression and severe infection types, such as pneumonia and CLABSI, further contributes to these elevated rates [11]. Our findings underscore that in this highly vulnerable population, characterized by prolonged ICU stays (median: 29 days) and advanced malignancy, the development of an HAI, particularly a respiratory infection, often represents a critical or terminal event rather than a manageable complication.

In our study, since the presence of an HAI was a defining characteristic of the study population, the primary determinants of mortality were the type of infection (particularly pneumonia and CLABSI) and the oncologic status. This discrepancy suggests that our cohort reflects a severely ill and highly selected ICU population characterized by high malignancy prevalence and prolonged stays. In a similar vein, Dabar et al. reported in a multicenter cohort study that patients with HAIs experienced significantly higher mortality rates than those with community‑acquired infections. Their findings identified healthcare‑associated origin, elevated Acute Physiology and Chronic Health Evaluation II (APACHE II) scores, and infections caused by multidrug‑resistant (MDR) *Pseudomonas* or fungal pathogens as independent predictors of mortality [12]. This discrepancy suggests that our cohort reflects a severely ill and highly selected ICU population characterized by high malignancy prevalence and prolonged stays. The median time to infection diagnosis of 24 days indicates that many of these patients had already transitioned into a state of chronic critical illness, characterized by persistent organ dysfunction and profound immune exhaustion. In such a vulnerable population, the development of a secondary HAI, particularly pneumonia, likely represents a terminal event rather than a manageable complication.

The longer ICU and hospital stay observed among survivors is at least partly attributable to survivorship bias. Patients who die earlier have fewer days available to accumulate length of stay, whereas survivors must remain alive long enough to reach discharge. This finding should not be misinterpreted as a protective effect of prolonged stay; rather, it reflects the inherent temporal difference in the clinical course between the two groups. This bias also affects infection epidemiology, as patients who survive longer inherently have more time at risk for developing HAIs, which may influence the distribution of infection types between survivors and non-survivors. Therefore, LOS-related comparisons and infection timing should be interpreted cautiously.

In our study population, pneumonia, comprising both VAP and HAP, emerged as the most prevalent type of healthcare-associated infection (45.1%, n = 117). Multivariable analysis further revealed that pneumonia was an independent predictor of ICU mortality, associated with an approximately threefold higher risk of death compared to other infection types (aOR: 2.89, 95% CI: 1.41-5.94, p = 0.004). These findings are consistent with data from both Europe and other regions [12-15]. In the 10-year observation in Southern Poland, pneumonia and bloodstream infections (BSI) were the most common HAI types, with a pneumonia incidence density of 15.2/1,000 ventilator days and a CLABSI density of 8.0/1,000 central venous catheter days [15]. Our data emphasize that pneumonia and CLABSI are not only drivers of morbidity but also powerful independent predictors of mortality. Dabar et al. also identified HAI origin, MDR *Pseudomonas*, and fungal infections as independent risk factors for hospital mortality, reinforcing our findings regarding the lethal nature of these specific infection types [12].

In our study, in an additional sensitivity analysis restricted to VAP cases only (excluding HAP), pneumonia remained independently associated with ICU mortality (aOR: 2.71, p = 0.007), indicating that the prognostic impact of pneumonia was not driven solely by the small HAP subgroup. While time-to-event analyses are often preferred in ICU research, the Cox model in our cohort did not identify significant independent predictors of mortality. This likely reflects the exceptionally high mortality rate and the relatively homogeneous timing of death among patients with prolonged ICU stays. In this context, the binary outcome (death versus survival) may represent a more clinically informative endpoint than the precise timing of death. Therefore, multivariable logistic regression was retained as the primary prognostic model.

Furthermore, an analysis of national surveillance systems in Taiwan, South Korea, and Japan by Chiang et al. reported a nearly 50% reduction in total HAI incidence between 2008 and 2015; however, pneumonia and BSIs, particularly device-associated forms, remained the most critical infection types in terms of clinical burden [14]. That report highlighted *Staphylococcus aureus*, *Pseudomonas aeruginosa*, *Klebsiella pneumoniae*, and *Acinetobacter baumannii* as the predominant pathogens in HAP/VAP [14]. In our study, the fact that the vast majority of pneumonia episodes were caused by high-virulence and often MDR pathogens such as *Klebsiella* spp. (44.5%, n = 61) and *A. baumannii *(33.6%, n = 46) provides a microbiological explanation for the high lethality of pneumonia.

An ecological analysis conducted in 21 Brazilian hospitals during the early phase of the COVID‑19 pandemic similarly documented a marked rise in CLABSI rates and a shift in the pathogen spectrum toward *Candida *sp. and *Enterococcus faecalis*. These findings underscore that device‑associated infections continue to be major contributors to mortality, particularly in settings where healthcare systems are under substantial strain [13].

Surveillance studies conducted in high-income countries have shown that HAI prevalence can be reduced over time. For example, in the comparison of two national point prevalence surveys conducted in the United States by Magill et al., the overall hospital HAI prevalence decreased from 4.0% in 2011 to 3.2% in 2015 [16].

This decline has been most notable in infection types such as ventilator‑associated pneumonia, catheter‑related bloodstream infections, and surgical site infections, and has been attributed to structured infection control initiatives. Contributing factors include enhanced hand hygiene adherence, more judicious use of invasive devices, and the implementation of surveillance‑driven feedback strategies. Nevertheless, the same report and up-to-date CDC data indicate that infections caused by opportunistic pathogens, especially resistant Gram-negative bacteria and *Candida* sp., remain a major concern in ICUs, leading to particularly high mortality in complex, elderly, and highly comorbid patient populations [16,17].

The EUROBACT study, which investigated healthcare-associated bloodstream infections in ICUs, drew attention to the relationship between resistance patterns of causative pathogens and clinical outcomes. In this study, which included 162 ICUs from 24 countries, approximately three-quarters of hospital-acquired bloodstream infections were ICU-acquired, and about half of the isolated microorganisms were MDR, while one-fifth were extensively drug-resistant (XDR). The 28-day mortality rate was reported as 35.7%, and infection with MDR pathogens was found to be independently associated with mortality; inadequate control of the infection source was identified as one of the strongest risk factors for death [5]. These findings demonstrate that, in addition to the type of pathogen and its resistance profile, timely and effective control of the infection focus is a key determinant of prognosis.

These results emphasize the need for aggressive infection prevention strategies, particularly for pneumonia, and suggest that prognostic models for oncology ICUs must prioritize both the site of infection and the presence of malignancy to accurately reflect patient risk.

A distinctive feature of our study is that it reflects the epidemiology of HAIs in a specialized oncology ICU that remained a non-COVID facility throughout the 2020-2024 period. This allowed for a focused analysis of prognostic factors in patients with cancer, free from the significant confounding effects of the COVID-19 pandemic on ICU case mix and mortality reported in many other contemporary studies.

Limitations

There are several limitations to the present study. First, its retrospective, single-center design may restrict the generalizability of our findings to other clinical settings and introduces the potential for residual confounding. Second, although the multivariable model demonstrated acceptable discrimination, the explained variance was modest, reflecting the highly complex and multifactorial nature of mortality in critically ill patients.

A primary limitation is the absence of standardized illness severity scores, such as APACHE II or SOFA. Due to the retrospective nature of the data, key physiological and laboratory variables were not consistently recorded at uniform time points, preventing a reliable calculation of these scores for the entire cohort. Consequently, our model could not formally adjust for baseline illness acuity. Nevertheless, the high proportion of patients requiring invasive mechanical ventilation and central venous catheterization suggests that the study population represented a uniformly high-acuity cohort.

Regarding our analytical approach, we focused on the first documented HAI episode per patient to provide an unbiased, patient-level assessment. While alternative strategies, such as selecting the most severe or the final infection episode, might yield different associations, our method ensures statistical independence. Furthermore, although causative microorganisms were identified, standardized MDR/XDR classification data were not consistently available across the five-year period due to evolving laboratory reporting practices. Therefore, antimicrobial resistance status could not be incorporated into the multivariable prognostic model.

The study is also subject to survivorship and time-dependent biases inherent to its design. Non-survivors often had a shorter "time at risk" to accumulate length of stay or develop subsequent infections. While we addressed this by using the first HAI episode and observed no significant difference in the "time-to-infection" between survivors and non-survivors, residual time-dependent confounding cannot be entirely excluded. Moreover, the relatively long interval between ICU admission and HAI diagnosis (median: 24 days) introduces the possibility of immortal time bias. Our findings may, therefore, specifically reflect the prognostic drivers of mortality in patients with chronic critical illness and may not be generalizable to those who experience early ICU mortality before an HAI manifests.

It is also important to emphasize that our analysis was strictly restricted to patients who developed at least one HAI. Consequently, the identified prognostic factors characterize the risk of death within this specific infected subgroup rather than the broader ICU population. Additionally, granular oncologic data, such as exact malignancy staging or the precise timing of recent chemotherapy/radiotherapy, were not integrated into the analysis. Finally, as the primary endpoint was ICU mortality, long-term outcomes, including 90-day survival and post-discharge functional status, were not assessed.

Despite these limitations, the five-year duration and the focus on a high-acuity population provide valuable insights into the prognostic determinants of healthcare-associated infections in this vulnerable cohort.

## Conclusions

This five-year analysis demonstrates that mortality is exceptionally high among oncology intensive care unit patients who develop healthcare-associated infections. In this cohort, older age, underlying malignancy, and pneumonia (including ventilator-associated and hospital-acquired pneumonia) were identified as the primary independent factors associated with ICU mortality. These associations should be interpreted in the context of a retrospective design, modest explained variance, and the absence of standardized illness severity scores. While CLABSI and other infection types also contributed to the clinical burden, pneumonia stood out as the most significant independent prognostic factor for death, likely reflecting the impact of prolonged ICU stays, device exposure, and the predominance of high-virulence pathogens such as *Klebsiella* spp. and *Acinetobacter baumannii*.

The observation that age and malignancy are associated with higher mortality risk suggests that clinical management and goals of care should account for the patients' underlying oncologic and physiological vulnerability, rather than implying direct causality. Our findings highlight the need for strengthened, site-specific infection prevention strategies, particularly for respiratory infections, and robust antimicrobial stewardship programs tailored to oncology-focused ICUs. Future studies should incorporate standardized illness severity metrics, detailed antimicrobial resistance profiles, and frailty indicators into prognostic models to better account for potential confounding and to refine risk stratification in this highly vulnerable population.
